# Research on cabbage transplanting status detection and operation quality evaluation in complex environments based on improved YOLOv10-TQ and DeepSort

**DOI:** 10.3389/fpls.2025.1726334

**Published:** 2025-12-11

**Authors:** Yiqun Wang, Mengchen Liu, Yue Geng, Junjie Zhu, Wenbai Chen, Chunjiang Zhao

**Affiliations:** 1 College of Automation, Beijing Information Science and Technology University, Beijing, China; 2 National Engineering Research Center for Information Technology in Agriculture, Beijing, China

**Keywords:** YOLO, DeepSORT, cabbage, transplanting, quality assessment

## Abstract

The quality of crop transplanting is a critical factor influencing both plant survival and final yield. However, in mechanized transplanting operations, manual inspection suffers from low efficiency, while traditional algorithms struggle with poor environmental adaptability and insufficient detection accuracy. To address these limitations, this study proposes a detection-and-tracking-based method for recognizing and counting cabbage transplanting states in open-field scenarios, enabling accurate identification and robust tracking of seedling transplanting conditions. An improved YOLOv10-TQ detection network is developed by integrating a triplet attention mechanism and a combined QFocal Loss–cross-entropy loss function to enhance the detection accuracy and stability for three transplanting states of cabbage: normal, soil-buried seedlings, and bare-root seedlings. In addition, a lightweight MobileViT feature extraction network is incorporated into the DeepSort algorithm to improve fine-grained target representation, and, combined with a line-crossing counting strategy, this approach enables identity de-duplication and robust counting performance. Experimental results demonstrate that the proposed method achieves a mean average precision (mAP) of 86.3% and an average counting accuracy of 97.8% on a self-constructed cabbage transplanting dataset. Based on this study, a visualization system for monitoring cabbage transplanting status was designed to enhance precision in agricultural practices. Compared with traditional detection and counting methods, the proposed approach exhibits significant advantages in detection accuracy, tracking stability, and counting precision, providing a promising technical foundation for intelligent quality evaluation of cabbage transplanting operations and data-driven decision-making in agricultural machinery systems.

## 1 Introduction

China is the world’s largest producer of vegetables, leading globally in both cultivation area and total output. Over 60% of vegetable crops in the country are cultivated using transplanting techniques (Lin et al., 2021). As a representative open-field vegetable, cabbage is widely grown through transplanting. Traditional transplanting relies heavily on manual labor, which is repetitive and labor-intensive, with low operational efficiency and high susceptibility to human error (Ghazal et al., 2024). Although mechanized transplanting has improved efficiency, challenges such as poor adaptability to diverse field conditions, limited generalizability of machine parameters, and frequent mechanical issues—such as seedling tray blockages—often result in substandard transplanting quality, including overly deep planting, insufficient depth, or missed seedlings (Sun et al., 2024). Currently, transplanting quality is predominantly assessed through manual inspection, which is inefficient, laborious, and conducted under suboptimal working conditions. This not only increases labor costs but also limits scalability in large-scale production and field trials under the paradigm of precision agriculture (Zhao et al., 2024; Cui et al., 2023). Therefore, achieving accurate identification and counting of cabbage transplanting states is crucial for evaluating transplanting quality and ensuring the effectiveness of mechanized operations.

Traditional manual counting methods rely on visual observation, which is time-consuming, labor-intensive, and inefficient. The visual similarity among field crops and the subjectivity of human judgment often lead to significant errors, particularly in large-scale operations, making such methods inadequate for the demands of precision agriculture. To address these limitations, a range of image processing-based counting methods has been developed. For example, Zhang et al. (2021a) proposed a method for fruit counting and organ classification in pomegranate trees under natural conditions, using multi-feature fusion and Support Vector Machines (SVM). While effective in identifying most organs, the method demonstrates limited accuracy in trunk recognition. Similarly, Liang et al. (2020) proposed a dynamic row-counting approach for cotton during the crop protection stage, based on Histogram of Oriented Gradient (HOG) features and SVM. By defining regions of interest (ROIs), extracting HOG features, and training SVM classifiers, the method achieved strong robustness against environmental variations such as lighting changes, camera shake, and wind, with recognition rates exceeding 90%. This provided valuable technical support for semi-structured row-planted crops. However, traditional image-based methods rely heavily on handcrafted features, which limit their generalization capability and robustness. Furthermore, they tend to suffer from high latency, over-sensitivity to environmental changes, and susceptibility to noise, restricting their applicability in complex field conditions.

In recent years, the rapid development of deep learning has provided new paradigms for crop detection and counting. Deep learning-based counting methods are generally classified into two categories: object detection and object tracking. Detection-based methods typically involve capturing images using a camera moving at a constant speed, with a predefined sampling frequency to avoid frame overlap. The total crop count is then estimated by multiplying the number of detections per frame by the total number of frames. However, detection-only approaches face inherent challenges in configuring camera speed and sampling frequency. Overlapping regions between frames can result in significant overcounting, while factors such as occlusion and illumination variations may lead to missed detections. As a result, detection-based counting alone often fails to provide reliable accuracy under complex field conditions.

To overcome these limitations, tracking-based approaches have gained increasing attention due to their ability to maintain object identity across frames. Object tracking-based counting maintains object identity across consecutive frames, effectively reducing duplicate counts. Tracking algorithms such as DeepSort and ByteTrack have gained widespread adoption in agriculture due to their robustness and consistent performance. Wu et al. (2023) proposed a multi-object tracking and counting method for wheat ears using UAV videos. By enhancing YOLOv7 with Omni-Dimensional Dynamic Convolution (ODConv), Global Context Network (GCNet), and Coordinate Attention (CA), and replacing the DeepSort feature extractor with an improved ResNet architecture, the method achieved a mean detection accuracy of 96.2% and a tracking accuracy of 75.4%, offering valuable support for wheat yield estimation.

Similarly, Lyu et al. (2023) developed a grape yield estimation method by integrating YOLOv5s with a self-correcting Non-Maximum Suppression (NMS) mechanism and ByteTrack. Their approach addresses object overlap caused by occlusion and volume expansion, as well as target loss due to unstable video. Through camera motion compensation and an improved Kalman filter, the system enables automatic counting with significantly improved accuracy compared to traditional ByteTrack implementations. Liu et al. (2024) designed a defect detection and counting system for dried Hami jujubes, combining an optimized YOLOv7 detector with ByteTrack for multi-object tracking. The system achieves high-precision detection and classification of randomly arranged jujubes, reaching a counting accuracy of 90.12%, thereby supporting automated grading and sorting processes. CottonSense, developed by Bolouri et al. (2024), is a high-throughput field phenotyping platform tailored for cotton boll segmentation and counting. Utilizing an RGB-D camera to capture both 2D and 3D data, and employing a Mask R-CNN framework based on ResNet and FPN, the system accurately segments cotton fruit across four growth stages (square, flower, closed boll, and open boll), with a segmentation accuracy of 79%. Combined with SORT-based tracking, it achieves a correlation of 0.93 with manual counting, providing robust technical support for cotton breeding and yield optimization. To facilitate the timely identification and removal of wilt-diseased pine trees, Ye et al. (2024) proposed a method combining an improved YOLOv5 with StrongSORT. By incorporating geometric features of infected trees, the approach enables visual tracking and counting in complex forest environments, achieving a detection accuracy of 92.4% and a tracking accuracy of 72.3%. Cui et al. (2023) presented a real-time missing seedling counting method for rice fields, leveraging a lightweight YOLOv5s detector integrated with ByteTrack. Enhancements include an optimized detection head, improved loss function, Transformer encoder for feature enhancement, and channel pruning for model compression. Experimental results show the method achieves a counting accuracy of 93.2%, with a fivefold increase in efficiency over manual counting, offering a practical and scalable solution for field-level seedling monitoring. To achieve rapid and accurate assessment of peanut pod appearance quality, Liu et al. (2025) developed a lightweight YOLOv5 SSE model that integrates ShuffleNetv2 and SE attention modules. The method enhances detection precision while reducing computational cost, achieving a mean average precision of 99.3% and a peak processing speed of 192.3 FPS.

In precision agriculture, counting plays a crucial role in providing temporal, spatial, and individual-level data, enabling systematic monitoring of crop growth, yield forecasting, and plant health assessment. These data-driven insights facilitate localized and informed decision-making for field management. While object tracking algorithms have demonstrated effectiveness in areas such as yield estimation, pest monitoring, crop surveillance, and phenotypic analysis, their application in transplanting scenarios remains underexplored. This highlights the pressing need for intelligent systems capable of evaluating transplanting quality in an automated and reliable manner. To address the limitations of manual assessment and the shortcomings of existing detection–tracking algorithms, particularly their difficulty in recognizing transplanting status and high counting errors, this study proposes a novel cabbage transplanting status recognition and counting method. By integrating an YOLOv10-TQ detection network with DeepSort tracking, the proposed approach enables accurate identification and counting of three distinct transplanting conditions in cabbage.

The main contributions of this work are as follows:

1. Definition of cabbage transplanting status categories and dataset construction: A standardized taxonomy of cabbage transplanting status is established, along with a dedicated dataset for model training and performance evaluation in detection and tracking.
2. Design of the YOLOv10-TQ detection framework: YOLOv10-TQ, a novel detection model is proposed, featuring an enhanced feature representation network with an integrated triplet attention mechanism. A joint QFocal Loss and cross-entropy loss function is introduced to address class imbalance, improving the model’s accuracy and robustness in detecting three transplanting states.
3. Development of a lightweight DeepSort-based counting algorithm: A tailored DeepSort tracking algorithm is designed for transplant counting. The feature extraction module is restructured using MobileViT XXS and optimized extraction ratios to enhance representation while maintaining computational efficiency. Furthermore, a line-crossing counting strategy is employed to eliminate duplicate counts and improve overall counting precision.

The remainder of this paper is organized as follows: Section 2 describes the materials and methods, including data collection, dataset preprocessing, and the deep learning algorithms employed. Section 3 presents the experimental setup and result analysis, with a series of comprehensive experiments conducted to evaluate the proposed approach. Section 4 provides the conclusions of the study.

## 2 Materials and methods

### 2.1 Data acquisition and dataset development

#### 2.1.1 Standardized classification criteria for cabbage transplanting status

**Figure 1:**
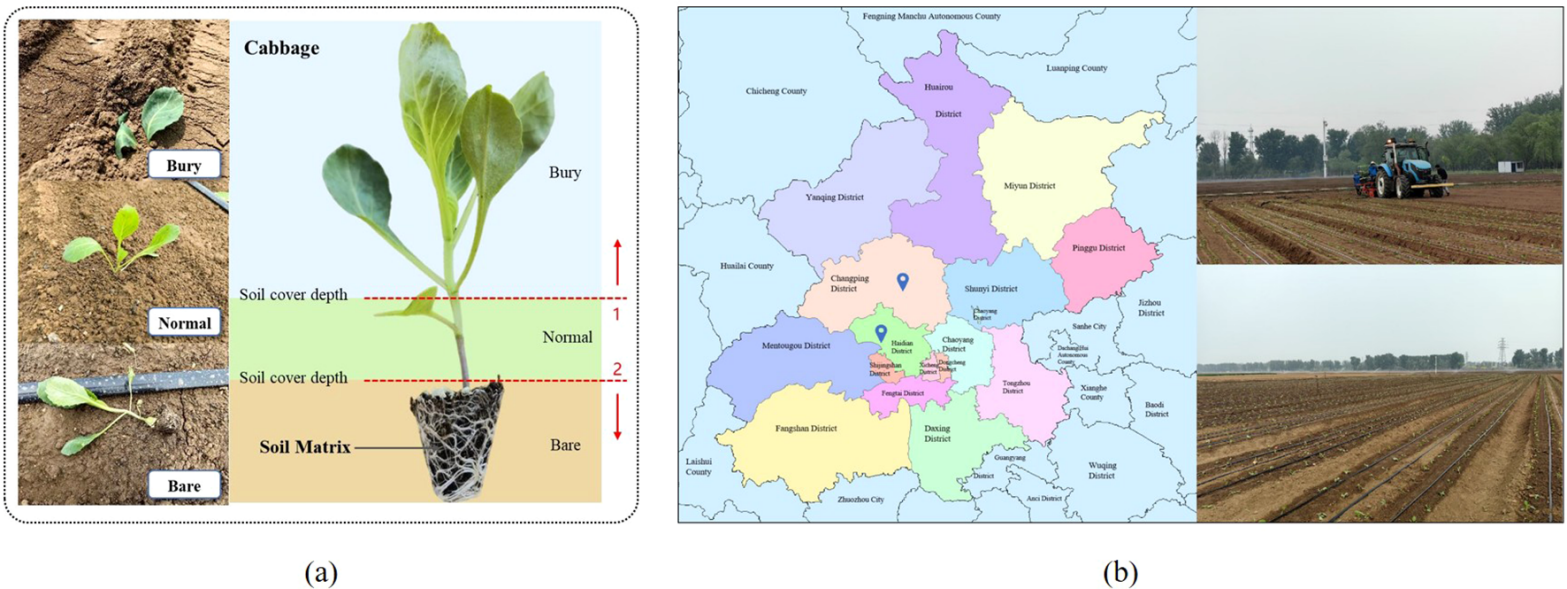
Cabbage transplanting overview. **(A)** Standardized classification of cabbage seedling transplanting status. **(B)** Transplanting locations and field environments.

The cabbage transplanting status is determined by the interaction among the transplanting mechanism, soil conditions, and surrounding environment. As illustrated in **Figure 1a**, cabbage plug seedlings are classified into three categories: normal, buried, and bare-root. In the normal status, the seedling is placed at an appropriate depth, where the root zone is adequately covered by soil substrate, and the leaves remain fully exposed above the surface. In the buried status, the seedling is transplanted too deeply, resulting in excessive soil coverage that partially or completely obscures the leaves. In the bare-root status, the seedling is placed too shallowly, leaving part or all of the root substrate exposed to air. In **Figure 1a**, the region above dashed line 1 indicates soil coverage exceeding the upper threshold, corresponding to the buried status. The region below dashed line 2 indicates soil coverage below the lower threshold, corresponding to the bare-root status.

#### 2.1.2 Dataset development and image preprocessing

As illustrated in **Figure 1b**, Image data were collected from two field sites: the Xiaotangshan National Precision Agriculture Research and Demonstration Base and the Taizhouwu Experimental Station in Beijing. The cabbage cultivar used was Zhonggan-21. Data collection was conducted to comprehensively capture illumination variations during the spring and autumn transplanting seasons (April 20–22, August 30–31, and September 13–14, 2024). The acquisition schedule covered multiple lighting conditions: (1) clear midday (11:00–13:00), characterized by intense sunlight, soil glare, and leaf shadows, representing strong-illumination scenarios on mulched plots; (2) overcast daytime (08:00–17:00), providing uniform lighting without distinct shadows, suitable for cloudy-field environments; (3) late afternoon (16:00–18:00), when solar intensity weakens and the incidence angle lowers, simulating low-light scenes before sunset; and (4) random rainfall periods, with dim lighting and reflective wet soil surfaces, representing the complex illumination conditions under rainy weather. A Huawei P60 smartphone was used for image acquisition, with still images captured at a resolution of 4000 × 3000 pixels and videos recorded at 3840 × 2160 pixels. To simulate real-world conditions such as uneven terrain, video streams were captured from multiple angles to reflect the practical variability of transplanting scenarios. In addition, close-up images of individual cabbage seedlings were collected to capture fine-grained visual features essential for transplanting status recognition.

The experimental plots featured light loam soil, optimal for cabbage cultivation, while also incorporating naturally occurring field variations such as soil clods formed by drought or mechanical compaction and mulched-surface soils. After transplanting, cabbages were arranged at a plant spacing of 35 cm and row spacing of 45 cm. The transplanting operations employed a Kubota 2ZSY-4 vegetable transplanter, operating at an average speed of 0.35ms^−1^, combining mulched-field and bare-soil transplanting methods. Some plots were covered with plastic mulch prior to transplanting, whereas others were directly transplanted on bare soil.

Following data cleaning and selection, 2,610 cabbage images were retained in JPG format and uniformly resized to 800 × 600 pixels. To increase dataset diversity and enhance the model’s generalization capability, 166 various data augmentation techniques were applied. The final dataset consisted of 6,871 images, which were randomly split into training, validation, and test sets with a ratio of 8:1:1.

#### 2.1.3 Cabbage tracking dataset development

In practical cabbage transplanting scenarios, environmental conditions are often complex and variable. To better reflect diverse field environments and comprehensively assess tracking performance, representative cabbage samples from multiple scenes were selected for annotation. To meet the requirements of field level counting, frame-by-frame annotations were performed on raw video data using the DarkLabel tool. Annotation information was stored in a customized CSV format: frame,cname,id,x1,y1,x2,y2, where each parameter denotes the frame number, class name, object ID, and the coordinates of the top-left and bottom-right corners of the bounding box, respectively. Based on the annotated ground-truth trajectories, individual cabbage plants were cropped from the video frames. The resulting dataset was split into training and validation sets with a ratio of 8:2. The final appearance dataset consisted of 158 distinct cabbage plants, totaling 15,479 images. On average, each plant contributed approximately 78 images to the training set and 20 images to the validation set.

**Figure 2:**
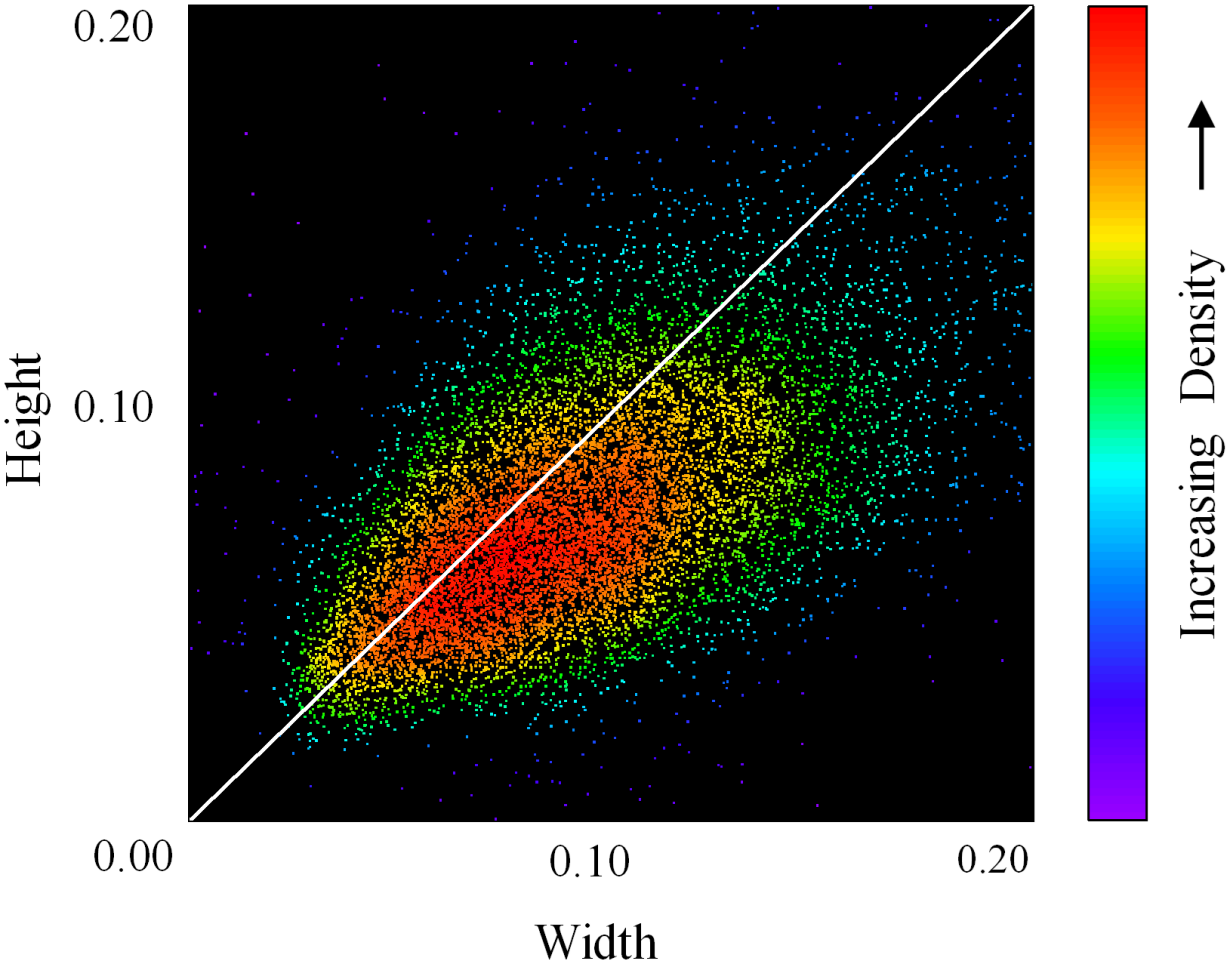
Width–height distribution of cabbage candidate bounding boxes.

The original re-identification network in the DeepSort algorithm was designed for pedestrian tracking. However, the motion patterns and visual characteristics of cabbage differ substantially from those of humans. Specifically, pedestrian feature extraction typically adopts a 1:2 aspect ratio, which is unsuitable for capturing the scale and appearance features of cabbage. As illustrated in **Figure 2**, the distribution of bounding box dimensions for cabbage is concentrated near a 1:1 ratio. Accordingly, a square aspect ratio (1:1) is adopted to better align with the geometric characteristics of cabbage and enhance feature extraction. To ensure compatibility with the network input while preserving the intrinsic visual features of the images, boundary padding is applied to the original three-channel images. A padding value of 255,255,255255, 255, 255255,255,255 is used (white background), and all images are standardized to a fixed resolution of 128 × 128 pixels.

### 2.2 Overall framework of the proposed method

**Figure 3:**
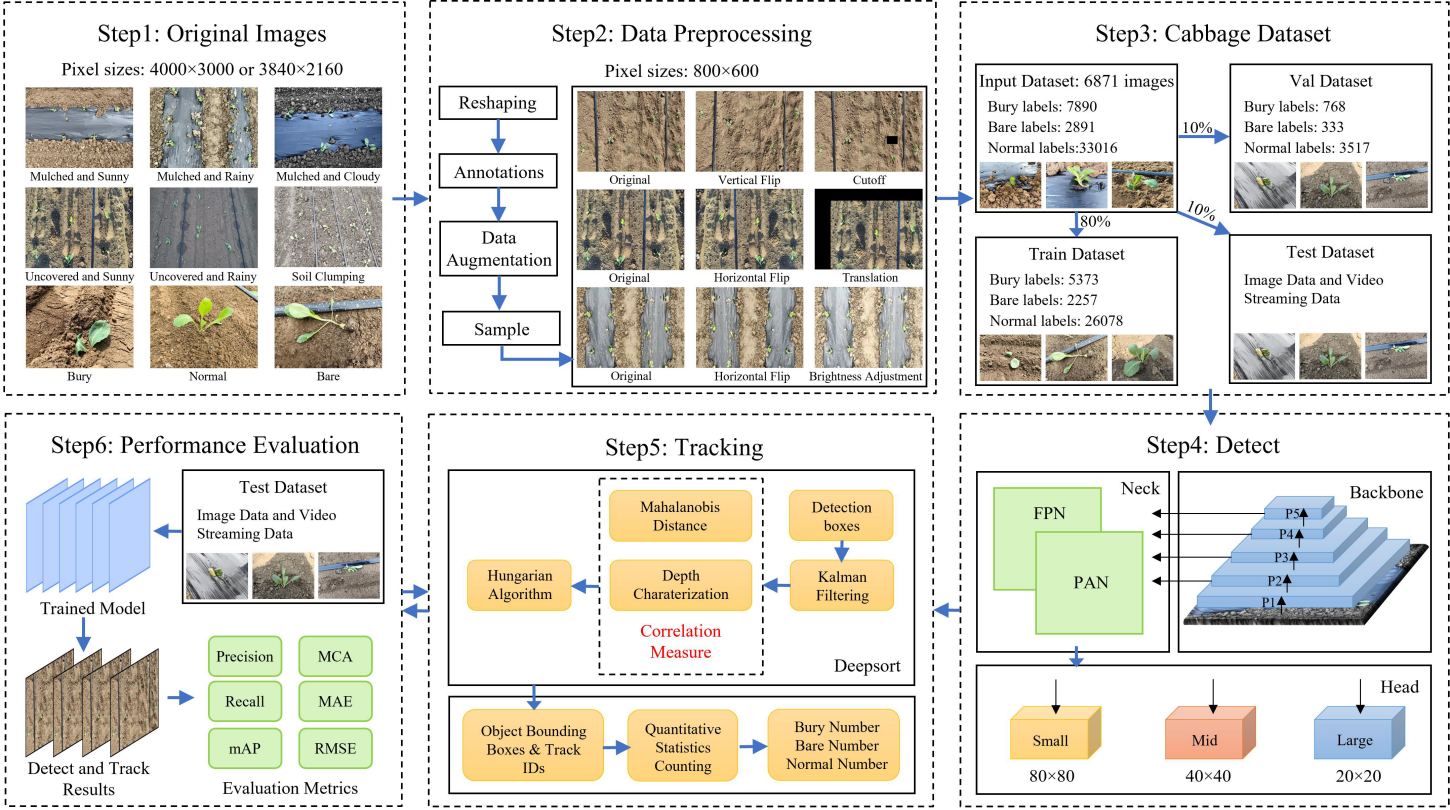
Schematic diagram of the proposed method.

The overall experimental framework for cabbage transplanting status detection and counting is illustrated in **Figure 3**. Considering that the quality evaluation of transplanting machine operations mainly relies on manual judgment, which is inefficient and unsuitable for large-scale operations, this study introduces a triplet attention mechanism based on YOLOv10 to enhance feature representation. A classification loss function combining QFocalLoss and cross-entropy loss is adopted to optimize detection performance. A lightweight real-time detection network is developed to identify transplanting states in cabbage images. The feature extraction network of DeepSort is replaced with the lightweight MobileViT XXS and retrained, which reduces the model’s computational cost while fully utilizing the fine-grained information in cabbage images. Finally, a line-counting method is introduced to achieve accurate counting of cabbage transplanting states.

### 2.3 The proposed YOLOv10-TQ detection network

In the research on cabbage transplanting state detection and counting, the tracking component relies on the detection results from the front-end to perform object matching and trajectory updates. Therefore, any high rate of false positives and missed detections during the detection phase will directly lead to confusion or even failure in tracking. Based on this, the YOLOv10 model is improved, and an enhanced YOLOv10-TQ algorithm is proposed. Specifically, a triplet attention mechanism is introduced to improve feature representation, and a classification loss function combining QFocalLoss and cross-entropy loss is employed to optimize detection performance. A real-time detection network is established to identify transplanting states in cabbage images, aiming to achieve more accurate and stable detection of cabbage transplanting states in complex environments, thus providing reliable judgment and real-time updates for subsequent object tracking.

#### 2.3.1 YOLOv10 object detection algorithm

**Figure 4:**
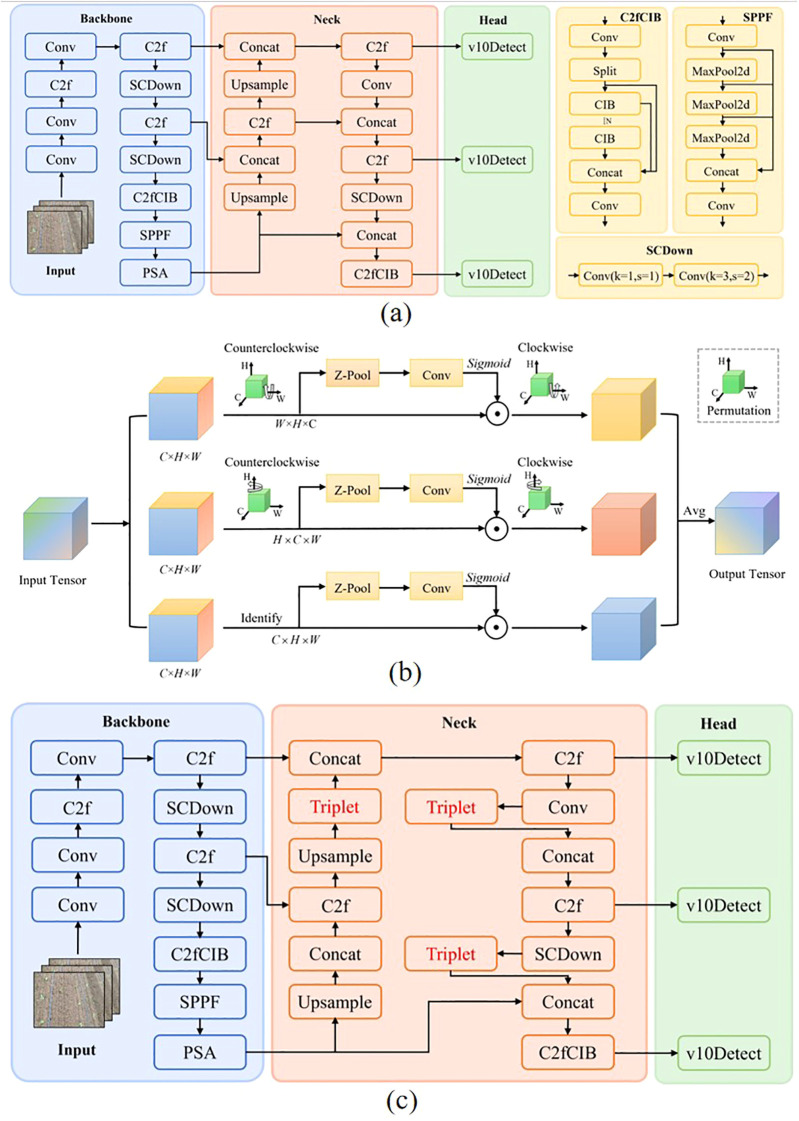
YOLOv10 and its improved architectures. **(A)** YOLOv10 network structure. **(B)** Improved YOLOv10 algorithm. **(C)** MobileOne block structure.

YOLOv10 (Wang et al., 2025) is one of the most advanced single-stage object detection algorithms to date. It introduces a Non-Maximum Suppression-Free (NMS-Free) design and incorporates model-level architectural optimizations to address limitations in post-processing and model structure present in previous versions, thereby achieving true end-to-end object detection (Nguyen et al., 2025). The YOLOv10 detection model consists of three main components: the backbone, the neck, and the head networks (Wu and Xu, 2025), as illustrated in **Figure 4a**. Building upon the convolutional layers (Conv), the C2f module—which emphasizes efficient feature aggregation—and the SPPF module for local-global feature fusion from YOLOv8, YOLOv10 further incorporates several structural innovations. These include the Spatial-Channel Decoupled Downsampling (SCDown) module, the C2fCIB module, and the Pyramid Split Attention (PSA) mechanism (Hu et al., 2025), which enhance multi-scale feature representation. The neck adopts an FPN-PAN structure to integrate feature information from different stages of the backbone. The head utilizes a lightweight decoupled design, separating classification, regression, and objectness prediction into distinct branches and aggregating their outputs. This architecture supports a unified dual-assignment strategy and aims to eliminate YOLO’s reliance on NMS during post-processing.

#### 2.3.2 Triplet attention mechanism

In the task of cabbage transplanting status detection, the complexity of field environments poses significant challenges for conventional attention mechanisms, which often fail to effectively capture critical features. Triplet Attention significantly differs from traditional attention mechanisms. As shown in **Figure 4b**, Triplet Attention can capture dependencies between multiple dimensions through rotational operations and residual transformations without requiring additional learnable parameters. At the same time, it integrates both channel and spatial information, thereby enhancing feature representation capabilities. Triplet Attention adopts a three-branch parallel architecture based on spatial attention. Two of the branches are designed to capture interactions between the channel and spatial dimensions, while the third branch focuses on modeling spatial attention. The outputs of all three branches are aggregated through a simple averaging operation (Yang et al., 2024). To enrich feature expression while reducing computational complexity, the Z-Pool layer fuses cross-dimensional average pooling (AvgPool) and max pooling (MaxPool), enabling effective dimensionality compression. The module accepts an input tensor and outputs a tensor of the same shape. In the first branch, the input tensor is rotated 90 degrees counterclockwise along the W axis to model interactions between the height and channel dimensions. This rotated tensor is then passed through the Z-Pool layer, a convolutional layer, and a Sigmoid activation function to compute the attention weights. It is subsequently rotated 90 degrees clockwise to align with the original input dimensions. The second branch performs a similar operation along the H axis to capture interactions between the width and channel dimensions. The third branch directly applies the Z-Pool layer, convolution, and Sigmoid activation to extract spatial dependencies from the input tensor.

Triplet Attention not only assigns weights to different feature channels of the input cabbage transplanting status tensor but also allocates weights to different spatial locations within each channel. This enables the network to extract cabbage features with finer granularity, enhancing the model’s capability for accurate target recognition. Triplet Attention was selected over CBAM, Coordinate Attention, and ECA-Net because it provides the best balance between accuracy and real-time efficiency for cabbage transplant status detection. Unlike CBAM’s Woo et al. (2018) sequential channel–spatial modeling and Coordinate Attention’s Hou et al. (2021) limited local encoding, Triplet Attention C. Zhang et al. (2021b) employs a three-branch parallel structure that simultaneously captures channel–space, space–channel, and spatial dependencies without additional parameters, preserving inference speed while enhancing fine-grained feature extraction for bare-root exposure and buried-seedling junctions. In contrast, ECA-Net Wang et al. (2020) focuses solely on channel dependencies and lacks spatial awareness, leading to localization errors in complex field scenes.

Therefore, in this study, a Triplet Attention module is integrated before each of the final three concatenation operations, as shown in **Figure 4c**. This design fully leverages the advantages of Triplet Attention to refine the processing of feature tensors, strengthen the allocation of weights across feature channels and spatial dimensions, and facilitate the precise extraction of critical cabbage features from complex field images, ultimately improving recognition accuracy.

#### 2.3.3 Loss function optimization

In industrialized transplanting scenarios, the continuous refinement of transplanting technologies has resulted in a limited availability of low-quality transplanting samples for evaluation tasks. Consequently, the available transplanting status datasets are typically small in scale and exhibit significant class imbalance. Focal Loss (Ross and Dollár, 2017), as a reweighting strategy, addresses this issue during training by introducing a modulating factor that reduces the dominance of frequently occurring, easily classified classes. Without such reweighting, easily classified negative samples tend to dominate the loss and gradient updates, which leads to insufficient learning from rare and hard examples. Given the inherent imbalance caused by the scarcity of minority class samples in transplanting status datasets, conventional Focal Loss partially alleviates the imbalance between positive and negative samples, but still struggles when dealing with complex or noisy samples. To further address this limitation, QFocalLoss extends the original Focal Loss by introducing a weight factor to better balance the distribution of positive and negative samples. This enhancement improves the model’s ability to learn from minority and hard examples, thereby increasing classification accuracy under imbalanced data conditions (Wang J. et al., 2023).

In the QFocalLoss function defined in Equation 1, *α_t_* in Equation 2 is used to balance the contributions of positive and negative samples, while the term |*y* − *σ*|*^γ^* in Equation 1 is designed to balance the contributions of easy and hard examples, where *y* denotes the ground-truth label and *σ* denotes the predicted probability. This design increases the loss function’s sensitivity to positive and high-quality samples, while reducing interference from redundant negative samples. However, although QFocalLoss offers a smoother optimization landscape compared to the original Focal Loss, it remains susceptible to noise and can concentrate excessive weight on mislabeled or noisy samples, thereby suppressing the loss contributions from easily classified instances. To mitigate this issue, QFocalLoss is combined with the binary cross-entropy loss defined in Equation 3 in a weighted manner, and the resulting formulation in Equation 4 is used as the classification loss function of the model. In this study, the weighting coefficients *j* and *k* in Equation 4 are both set to 0.5, allowing a balanced integration of the robustness of cross-entropy and the adaptive reweighting capability of QFocalLoss.

(1) $$Loss_{QFL}=-\alpha_{t}*|y-\sigma {|}^{\gamma }*[(1-y)*\text{log}(1-\sigma )+y*\text{log }(\sigma )]$$

(2) $$\alpha_{t}=y*\alpha +(1-y)*(1-\alpha )$$

(3) $$Loss_{BCE}=-(y*\text{log }(\sigma )+(1-y)*\text{log }(1-\sigma ))$$

(4) $$L_{cls}=j\times Loss_{QFL}+k\times Loss_{BCE}$$

### 2.4 DeepSort algorithm improvement

In the cabbage plant counting task, accurately tracking the same target across consecutive frames is critical to ensuring counting precision. Due to the low intra-class variability and high visual similarity among cabbage seedlings, it is necessary to assign distinct IDs to individual plants for effective trajectory tracking. Re-identification (ReID) techniques are introduced into tracking algorithms to extract discriminative features from targets and compute feature distances, which helps determine whether targets across different frames belong to the same entity. The DeepSORT algorithm (Wojke et al., 2017; He et al., 2016) builds upon the SORT framework by integrating a Kalman filter and the Hungarian algorithm, along with a ReID-based feature extraction network and a cascade matching strategy. This approach jointly considers both motion and appearance features of the targets. Although DeepSORT enhances SORT by incorporating deep feature representations, it still has limitations. For example, its feature extraction network is relatively shallow, which restricts its representation capability, and it does not fully exploit inter-channel relationships within the features. In the context of cabbage transplanting status counting, temporary target loss may occur due to occlusion or complex field conditions. To address this, the ReID module extracts deep features from the bounding boxes of cabbage seedlings, assisting the algorithm in re-identifying and re-associating previously tracked targets.

**Figure 5:**
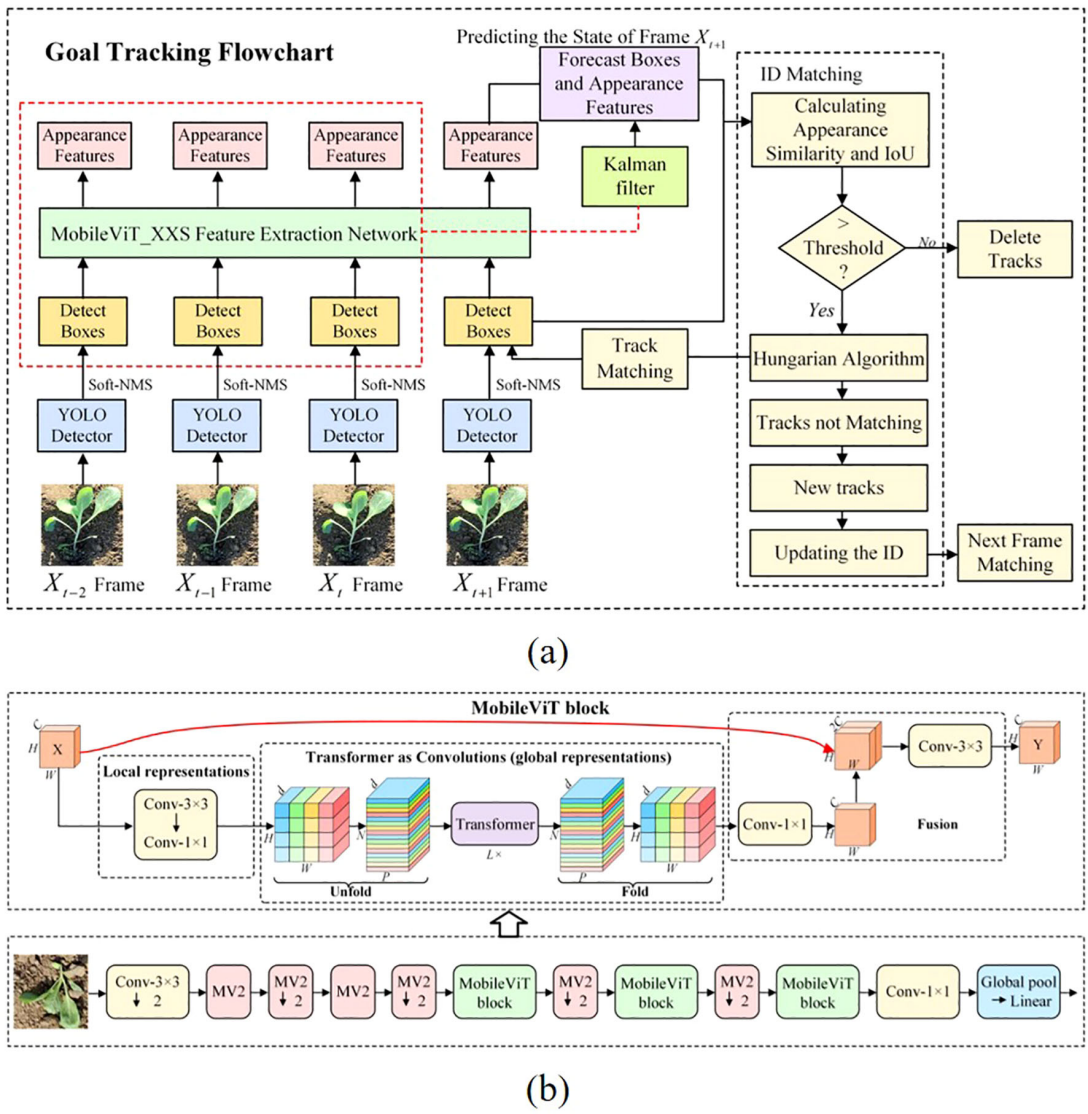
Overview of the object tracking and feature extraction pipeline. **(A)** Object tracking flowchart. **(B)** MobileViT network structure diagram.

As illustrated in **Figure 5a**, The tracking process is as follows: the bounding boxes of cabbage seedlings detected by the YOLOv10-TQ model are first obtained. Next, features are extracted for each tracked target, and similarity metrics—including feature similarity and Intersection over Union (IoU)—are computed between adjacent frames. If a match is successful, the Kalman filter updates the target’s state and predicts its future position and velocity. The matched data are then associated and assigned a unique ID. If a target remains unmatched for a number of frames exceeding a predefined threshold, its tracking information is removed.

#### 2.4.1 MobileViT feature extraction

MobileViT (Mehta and Rastegari, 2021) is a lightweight neural network proposed by Apple in 2021 that integrates Convolutional Neural Network (CNN) with Transformer architectures. It is designed to achieve joint modeling of local and global representations with a relatively small number of parameters. This design retains the efficiency and speed advantages of CNNs while incorporating the global contextual awareness characteristic of Vision Transformers (ViTs). MobileViT primarily consists of standard convolutional layers, MV2 blocks, and MobileViT blocks, as illustrated in **Figure 5b**. The MV2 block, which forms the foundation of the MobileViT architecture, is inspired by the inverted residual structure introduced in MobileNetV2. It first expands and then compresses the feature map dimensions, and applies residual connections between the input and output along the channel dimension. This structure preserves the nonlinearity and expressive power of the feature maps while maintaining parameter efficiency and computational effectiveness, thereby enhancing overall network performance.

The MobileViT BLOCK consists of three main components: a convolution-based local feature extraction module, a Transformer-based global feature extraction module, and a feature fusion module. For an input tensor *x* of *C* ×*H* ×*W* defined in Equation 5, where *x* denotes the input feature map and *C*, *H* and *W* denote the number of channels, height, and width, respectively, the MobileViT BLOCK first applies convolutions with kernels 3 × 3 and 1 × 1 to extract local spatial information and obtain the local feature map *X_L_* × *H* × *W* × d as in Equation 6, while mapping the input features to a higher-dimensional space. The Unfold operation, analogous to the data processing step in Transformers, converts *X_L_* into *N* non-overlapping sequence blocks *X_U_* × *P* × *N* × d, as formulated in Equation 7, and the Transformer Encoder then processes these blocks in parallel to perform global feature extraction and produce the global representation *X_G_*. Subsequently, the Fold operation reconstructs the features into *X_F_* × *H* × *W* × d, as given in Equation 8. Here, *P* = *W* × *H* and *N* = *H* × *W* × *P* denote the patch size and the number of sequence blocks, respectively, and are defined in Equation 9 and 10. A convolution with kernel 1 × 1 is then applied to *X_F_*, after which the resulting feature map is concatenated with the original input tensor *x* and passed through a convolutional layer with kernel 3 × 3 to produce the final feature map *Y*, i.e., the output of the MobileViT BLOCK that integrates both local and global information. This architecture, summarized in Equations 5–10, enables the MobileViT BLOCK to simultaneously leverage spatial inductive bias and global context, thereby enhancing the model’s capability to extract rich features from input data.

(5) $$X=R^{H\cdot W\cdot C}$$

(6) $$X_{L}=R^{H\cdot W\cdot d}$$

(7) $$X_{U}=R^{P\cdot N\cdot d}$$

(8) $$X_{F}=R^{H\cdot W\cdot d}$$

(9) P=W×H

(10) $$N=\frac{H\times W}{P}$$

Where, *C* represents the dimensions of the tensor, *H* refers to the height of the tensor, and *W* denotes the width of the tensor.

The original feature extraction network in DeepSORT consists of a simple stack of CNN layers, which fails to fully capture the fine-grained details of cabbage seedlings. MobileViT, on the other hand, offers both local and global advantages, making it more suitable for capturing the intricate features of cabbage. Considering the richness of feature extraction and the trade-off between model complexity and size, MobileViT_XXS is employed for feature extraction in the cabbage recognition task.

Under an equivalent lightweight configuration, MobileViT-XXS integrates local texture modeling (CNN) and long-range dependency learning (ViT), making it more robust to repetitive leaf-vein textures, slender contours, occlusions, and strong reflections typical in cabbage-field environments. With only about 1.3 M parameters, it achieves real-time inference on edge devices while maintaining higher accuracy than CNN-based alternatives (Mehta and Rastegari, 2021). In contrast, MobileNetV3 Howard et al. (2019) lacks explicit global dependency modeling, EfficientNet-Lite Tan and Le (2019) relies on deeper stacking to approximate long-range context, and ShuffleNet Ma et al. (2018), though extremely efficient, has a lower accuracy ceiling under texture ambiguity.

#### 2.4.2 Line counting method

When using the DeepSORT algorithm to track different transplanting states of cabbage, ID switching still occurs, particularly as the cabbage seedlings enter the camera’s field of view and undergo significant appearance changes. These variations often result in frequent ID reassignment, which leads to overcounting if ID information is used directly for counting purposes. Consequently, using the raw ID output from DeepSORT as a basis for transplanting state statistics introduces significant error in the total count. To address this issue of duplicate counting, a virtual counting line–based method is proposed.

**Figure 6:**
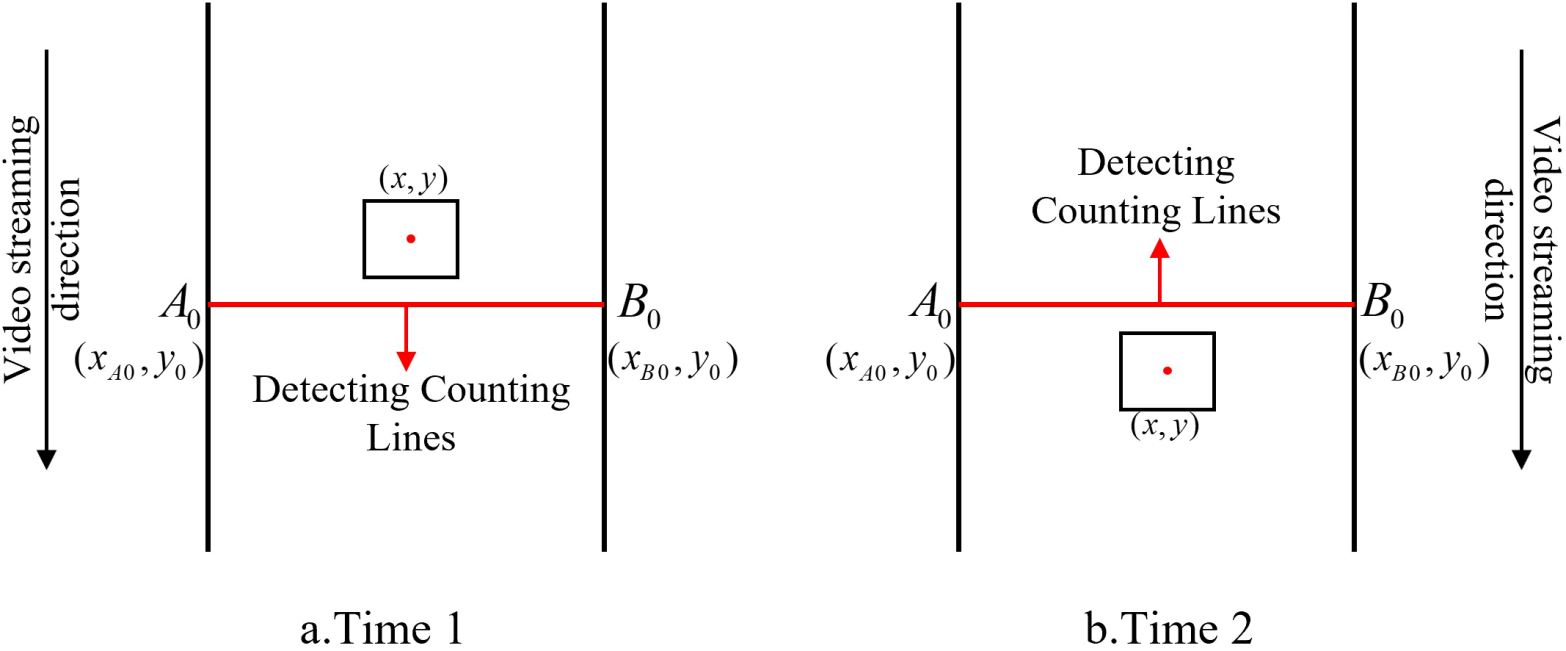
Line counting method.

As illustrated in **Figure 6**, the rectangle represents a cabbage seedling associated with a specific tracking ID, and the red dot denotes the center of its bounding box with image coordinates (*x,y*), where *x* and *y* are the horizontal and vertical coordinates of the center point, respectively. The red segment *A*_0_*B*_0_ represents the virtual counting line; its endpoints are A_0_=(*x_A_*_0_*,y*_0_) and *B*_0_=(*x_B_*_0_*,y*_0_), where *x_A_*_0_ and *x_B_*_0_ are the horizontal coordinates of the two endpoints and *y*_0_ is the vertical coordinate of the counting line. For a given tracking ID, a transplanting state is counted only when the corresponding seedling satisfies the following crossing condition: at time point 1, the center coordinate *y* is less than the line coordinate *y*_0_ (*y < y*_0_), and at time point 2, *y* exceeds *y*_0_ (*y > y*_0_). In other words, the virtual-line-based counting module updates the count for the transplanting state associated with that ID only when the center point moves from one side of the line to the other. This strategy effectively reduces duplicate counting of transplanting states.

## 3 Experiments and evaluation

### 3.1 Environment and hyperparameter configuration

The experiments were conducted on a deep learning framework built with Python 3.8.0 and PyTorch 1.9.0. The hardware environment consisted of an RTX 3090 GPU with 24 GB of memory.

#### 3.1.1 Hyperparameter configuration of the detection model

For the training of the detection model, the number of training epochs was set to 100, and the batch size was set to 16. the Adam optimizer was employed with an initial learning rate of 0.01.

The initial learning rate was set to 0.01 and the number of epochs to 100, considering the sensitivity of the YOLO-series detection head to learning-rate adjustments. A higher initial rate (e.g., 0.02) tends to cause oscillations in anchor-box matching parameters, while a lower rate (e.g., 0.005) prolongs the convergence process. The chosen initial rate of 0.01, combined with cosine-annealing decay (down to a minimum of 1 × 10^−5^), enables the model to rapidly approach the optimal solution in the early stages and to fine-tune weight parameters during later iterations Zhang et al. (2025). The epoch number of 100 was determined based on preliminary experiments, where the validation mAP stabilized at 86.3% after 60 epochs; extending training to 100 epochs helped prevent overfitting and fully optimize small-object detection parameters.

A batch size of 16 was selected in light of the dataset’s scale, representing a typical small-sample agricultural detection scenario. When the batch size was reduced to 8, insufficient sample diversity per iteration led to poorer generalization under special illumination conditions such as mulch reflections. Conversely, increasing the batch size to 32 accelerated convergence but caused GPU memory usage on the RTX 3090 (24 GB VRAM) to exceed 85%, while introducing risks of batch normalization statistical bias Su et al. (2025).

The Adam optimizer was adopted to effectively capture fine-grained features—such as seedling root exposure and leaf posture—in cabbage transplanting detection. By employing adaptive moment estimation (*β*_1_ = 0.9) and adaptive learning-rate scaling (*β*_2_ = 0.999), Adam mitigates gradient oscillations during small-batch training, making it particularly well-suited for small-sample agricultural visual tasks Wu et al. (2024). Furthermore, the lightweight design of YOLOv10 reduces model parameter complexity, and the parameter-wise update mechanism of Adam facilitates efficient weight optimization, preventing gradient vanishing in critical feature dimensions Wang et al. (2024).

#### 3.1.2 Hyperparameter configuration of the feature extraction model

For the feature extraction model, the Stochastic Gradient Descent (SGD) optimizer was used with an initial learning rate of 0.1. The batch size was set to 64, and the training was conducted for 40 epochs.

The initial learning rate was set to 0.1 and the total number of epochs to 40, considering that Transformer-based architectures require a relatively higher initial learning rate to effectively activate attention feature mappings. The value of 0.1 follows the “warm-up–decay” strategy of MobileViT, where the learning rate linearly increases to 0.1 over the first five epochs and then exponentially decays during the subsequent 35 epochs S. Wang et al. (2023). The epoch count of 40 was determined from preliminary experiments, which showed that the model loss converged to 0.21 after 20 epochs; extending training to 40 epochs further refined feature-matching parameters without overfitting, with the validation accuracy remaining stable at 97.1%.

A batch size of 64 was chosen considering the larger feature extraction dataset (15,479 images) and the requirement of Transformer blocks in MobileViT to update attention weights with larger sample batches for stable feature distribution. According to the linear scaling rule implemented in MindCV Zhao et al. (2021), the learning rate was proportionally adjusted to the batch size. A batch size of 64 allowed efficient convergence on a single GPU (≈ 14GB VRAM) without the need for distributed training.

The SGD optimizer was selected to ensure robust feature representation of cabbage seedling textures and morphological details. Although SGD lacks adaptive learning rate adjustment, it provides superior convergence stability in lightweight Transformer models. This stability effectively suppresses feature fluctuations caused by mulch reflections and soil noise in agricultural imagery, thereby mitigating overfitting and enhancing model generalization Zeiler and Fergus (2014).

### 3.2 Evaluation metrics

The evaluation metrics for object detection included Precision (*P*), Recall (*R*), mean Average Precision (mAP), and *F*1-Score.

(11) $$P=\frac{TP}{TP+FP}$$

(12) $$R=\frac{TP}{TP+FN}$$

(13) $$AP=\int_{0}^{1}P(r)\;dr$$

(14) $$\text{mAP}=\frac{1}{n}\sum_{i=1}^{n}AP_{i}$$

(15) $$F_{1}=\frac{2\times P\times R}{P+R}$$

Specifically, Precision and Recall are computed according to Equations 11 and 12, where *TP* (True Positive) refers to the number of instances correctly classified as positive, *FP* (False Positive) denotes the number of instances incorrectly classified as positive, and *FN* (False Negative) indicates the number of instances incorrectly classified as negative. The Average Precision (AP) and mAP metrics are obtained as in Equations 13 and 14, while the *F*1-Score is calculated following Equation 15, which balances Precision and Recall to evaluate the overall detection performance.

For evaluating the counting performance of the cabbage transplanting state statistics algorithm, three metrics were adopted: Root Mean Square Error (RMSE), Mean Absolute Error (MAE), and Mean Counting Accuracy (MCA), which are defined in Equations 16–18. RMSE is computed according to Equation 16 and reflects the deviation between the predicted count $\hat{y}_i$ and the ground truth $y_i$, with smaller RMSE values indicating better model performance. MAE, given in Equation 17, measures the average absolute difference between the predicted and true counts; a lower MAE indicates higher prediction accuracy. MCA, formulated in Equation 18, assesses the consistency between the predicted and actual counts of cabbage transplanting states, where a higher MCA indicates a closer match and thus better overall counting performance.

(16) $$\text{RMSE}=\sqrt{\frac{1}{n}\sum_{i=1}^{n}{\left(y_{i}-{\hat{y}}_{i}\right)}^{2}}$$

(17) $$\text{MAE}=\frac{1}{n}\sum_{i=1}^{n}|y_{i}-{\hat{y}}_{i}|$$

(18) $$\text{MCA}=\frac{1}{m}\sum_{i=1}^{m}\frac{y_{i}}{{\hat{y}}_{i}}\times 100\%$$

Where, *γ_i_* denotes the total number of cabbage transplanting state categories. *i* represents the *i*-th category of cabbage transplanting state. ${\hat{y}}_{i}$indicates the ground truth quantity for that transplanting state, while *y_i_* refers to the number of cabbage instances obtained through the counting algorithm. $\bar{y}$ represents the average number of cabbage transplanting states across all categories.

### 3.3 Loss function parameter selection

To determine appropriate values for parameters *γ* and *α* in the loss function, multiple configurations were tested and compared against the default settings. The experimental results are presented in **Table 1**. When *γ* =2 and *α* =0.3, although the model achieved the highest precision, it yielded the lowest recall and mean average precision (mAP). Notably, the detection accuracy for the bare-root transplanting state was significantly lower, indicating that the model struggled to identify bare-root seedlings accurately. This could lead to a substantial number of false negatives for this category, thereby undermining the overall evaluation of transplanting conditions—making it unsuitable for practical application in cabbage transplanting detection. When *γ* =1.5 and *α* =0.4, the model achieved the highest recall of 82.3%, but at the cost of lower precision. Specifically, the detection accuracy for the buried-seedling state dropped to 78.5%, indicating poor performance in identifying this condition, with a higher risk of false positives or missed detections.

**Table 1:** Comparison of parameter experiments, module ablation, and detection networks.

Experiment	Model/Config	Triplet attention	QFocalLoss_BCE	P (%)	R (%)	mAP (%)	AP (Normal)	Weights (MB)/GFLOPs/Params (M)
Parameter Experiment	*γ* =1.5, *α* =0.25	–	–	82.1	81.1	85.5	94.7	-/-/-
	***γ*** =**1.5, *α* =0.3**	–	–	**84.8**	**81.5**	**86.1**	**95.2**	-/-/-
	*γ* =1.5, *α* =0.4	–	–	82.1	82.3	84.8	94.6	-/-/-
	*γ* =2.0, *α* =0.3	–	–	86.2	78.3	84.6	95.0	-/-/-
	*γ* =1.0, *α* =0.3	–	–	84.3	79.2	85.6	94.9	-/-/-
Module Ablation	YOLOv10s	×	×	80.9	79.3	84.5	–	16.5/-/8.0
	Scheme1	✓	×	82.9	81.1	85.3	–	16.5/-/8.0
	Scheme2	×	✓	84.8	81.5	86.1	–	16.5/-/8.0
	Scheme3	✓	✓	85.3	82.1	86.3	–	16.5/-/8.0
Network Comparison	RT-DETR	–	–	81.2	78.1	83.7	–	40.5/56.6/40.5
	YOLOv8s	–	–	84.3	79.1	85.9	–	22.5/28.4/11.2
	YOLOv9s	–	–	81.7	77.7	84.3	–	15.2/26.7/7.2
	YOLOv10s	–	–	80.9	79.3	84.5	–	16.5/24.7/8.1
	YOLOv11s	–	–	82.1	78.6	85.4	–	19.2/21.3/9.4
	**YOLOv10s-TQ**	–	–	**85.3**	**82.1**	**86.3**	**-**	**16.5/24.7/8.1**

The bold values represent the highest mAP value under the most balanced trade-off between precision and recall.

In contrast, the configuration *γ* =1.5 and *α* =0.3 achieved the highest mAP of 86.1%, along with the most balanced trade-off between precision and recall. It also provided consistently strong performance across all three transplanting state categories. Therefore, *γ* =1.5 and *α* =0.3 were selected as the optimal parameters for QFocalLoss to ensure robust and balanced detection across different cabbage transplanting states.

### 3.4 YOLOv10 ablation experiment

To evaluate the contribution of each improvement to the YOLOv10 model, ablation experiments were 460 conducted on the test set, and the results are summarized in **Table 1**. By incorporating the Triplet Attention 461 module in Scheme 1, the accuracy improves to 82.9%, recall to 81.1%, and mAP to 85.3%, compared to the baseline model. This enhancement is achieved without significantly increasing the model complexity, effectively boosting the model’s ability to focus on features and improving both detection accuracy and comprehensiveness. In Scheme 2, the conventional classification loss function was replaced with the QFocalLoss BCE function. This adjustment further improved the model’s precision to 84.8%, recall to 81.5%, and mAP to 86.1%, effectively addressing the issue of class imbalance and enhancing detection robustness under complex field conditions. Scheme 3 combined both the Triplet Attention module and the QFocalLoss BCE function. This integrated approach yielded the best overall performance, with precision, recall, and mAP reaching 85.3%, 82.1%, and 86.3%, respectively. The results highlight a clear synergistic effect between the two enhancements, while keeping the model size and parameter count nearly unchanged, thus achieving optimal detection performance.

### 3.5 Comparison of different detection networks

To comprehensively validate the superiority of the proposed YOLOv10-TQ network over other object detection algorithms, a comparative experiment was conducted using the same cabbage transplanting status dataset while keeping all other training parameters constant. The dataset was respectively input into RT-DETR, YOLOv8s, YOLOv9s, YOLOv10s, and YOLOv11s models for evaluation. As shown in **Table 1**, the RT-DETR model exhibited the weakest performance across multiple metrics, achieving the lowest mAP of only 83.7% among all six models. Moreover, RT-DETR had the highest model complexity, with a weight size of 40.5 MB and GFLOPs reaching 56.6, indicating substantial computational and storage demands without delivering adequate accuracy and efficiency. Therefore, it is not well-suited for cabbage transplanting status detection tasks. YOLOv10-TQ enhances the fine-grained capture of soil coverage gradient (buried seedlings) and substrate exposure features (bare root) through the multi-dimensional feature interaction of the Triplet Attention mechanism. Combined with the QFocalLoss-cross-entropy joint loss function, it effectively mitigates the class imbalance issue. As a result, the improved model achieves an mAP of 86.3% under complex field backgrounds. Compared to YOLOv8s, YOLOv9s, YOLOv10s, and YOLOv11s, the mAP is higher by 0.4, 2.0, 1.8, and 0.9 percentage points, respectively, enabling more accurate differentiation of multiple transplant states, including normal transplant, bare root, and buried seedlings. Although YOLOv9s achieved the smallest parameter size, it had the lowest recall rate at only 77.7%. In contrast, YOLOv10-TQ reached a recall rate of 82.1%, indicating a stronger capability to detect actual targets in complex and dynamic cabbage transplanting environments and reduce missed detections. Therefore, the proposed YOLOv10-TQ model demonstrates a significant performance advantage in the detection module. It achieves a favorable balance between computational efficiency and detection accuracy, better meeting the operational requirements of transplanting quality assessment.

To further verify the robustness and statistical significance of the performance improvement achieved by the proposed YOLOv10-TQ, comparative experiments were conducted against baseline models, including YOLOv8s, YOLOv9s, YOLOv10s, and YOLOv11s, under identical training and evaluation settings. As shown in **Table 2**, YOLOv10-TQ achieves the highest mean Average Precision (mAP_0_._5_ = 86.3%), exceeding YOLOv10s by 1.8%.

**Table 2:** Statistical comparison of detection models.

Model	mAP0.5 (%)	95% CI (%)	P-value (*vs*. YOLOv10s)	Significance
YOLOv8s	83.7	[82.4 – 84.9]	*<* 0.01	Significant
YOLOv9s	84.3	[83.0 – 85.5]	0.027	Significant
YOLOv10s	84.5	[83.2 – 85.7]	—	—
YOLOv10-TQ (Ours)	**86.3**	**[85.1 – 87.6]**	**0.032**	**Significant (p *<* 0.05)**
YOLOv11s	85.4	[84.2 – 86.5]	0.041	Significant

The bold values represent the highest mAP value under the most balanced trade-off between precision and recall.

To ensure that this improvement is not a result of random variation due to the relatively small test set, we performed statistical significance testing. A paired *t*-test was conducted using five independent experimental runs for each model, and the 95% confidence intervals (CIs) for mAP were calculated. The results confirmed that the 1.8% mAP gain of YOLOv10-TQ over YOLOv10s is statistically significant (*p* =0.032 *<* 0.05), with a 95% CI of [0.6%, 3.0%]. Similarly, YOLOv10-TQ outperformed YOLOv9s and YOLOv11s with significant margins (*p <* 0.05), as summarized in **Table 2**.

These results demonstrate that the observed improvements are statistically reliable, not merely numerical fluctuations. The results validate the effectiveness of the proposed TQ module in enhancing detection accuracy and generalization, especially under complex transplanting conditions such as occlusion, soil reflection, and uneven lighting.

### 3.6 Cabbage detection visualization analysis

Grad-CAM (Gradient-weighted Class Activation Mapping) is a visualization technique used to interpret the decision-making process of deep learning models in classification tasks. By generating class activation maps, Grad-CAM provides a clearer understanding of the model’s attention and weight distribution across different regions of the input image. Grad-CAM++ optimizes the original Grad-CAM method by incorporating higher-order gradient information to weight the feature maps, enabling the capture of finer pixel-level details. In these maps, areas colored in red indicate regions that the model pays the most attention to, demonstrating high discriminative power. Yellow regions reflect areas of moderate attention, while blue regions represent areas with minimal relevance to the model’s decision-making process. These blue areas generally correspond to redundant information that has less impact on the cabbage transplanting state recognition.

**Figure 7:**
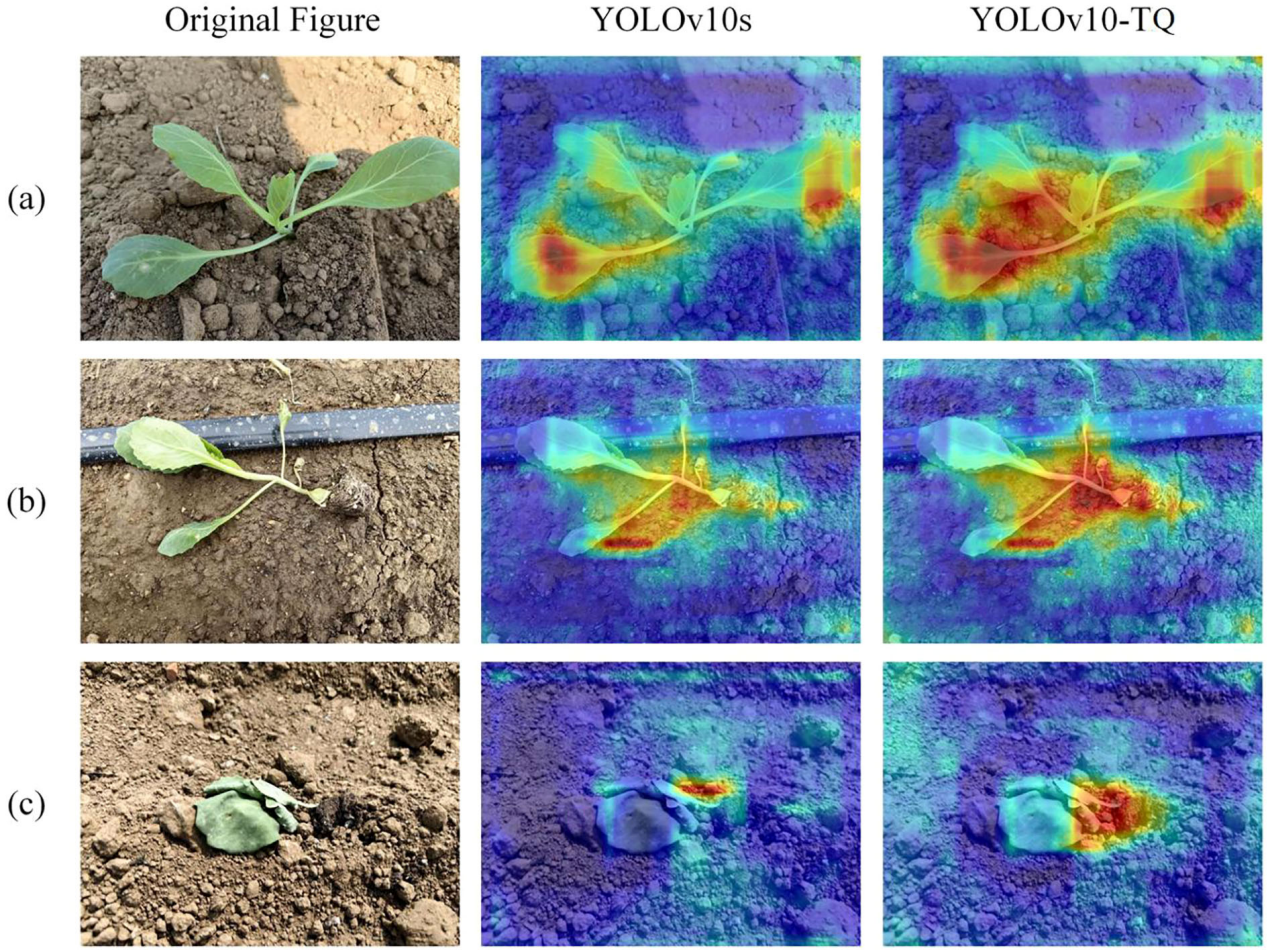
**(a)** Normal seedling; **(b)** Barried seedling; **(c)** Bare root.

**Figure 7** presents the Grad-CAM++ heatmaps before and after the improvement of YOLOv10. In **Figure 7a**, the model’s response to normal state cabbage is primarily concentrated around the leaf region. In **Figure 7b**, which shows the bare-root detection, the YOLOv10-TQ model places greater emphasis on the soil substrate compared to the original model. This enhancement effectively improves the accuracy of bare-root detection, allowing the model to more accurately capture root features in complex environments, and distinguish the different transplanting states of cabbage. In **Figure 7c**, while in the buried seedling state, the model’s attention is centered at the junction between the leaf and the soil. Compared to the YOLOv10s model, the YOLOv10-TQ model exhibits deeper color intensity and a higher response at the cabbage target, enabling more precise identification and focus on the subtle differences in transplanting states. This refined focus allows the model to better differentiate cabbage transplanting states, maintaining excellent detection performance even in diverse background settings.

**Figure 8:**
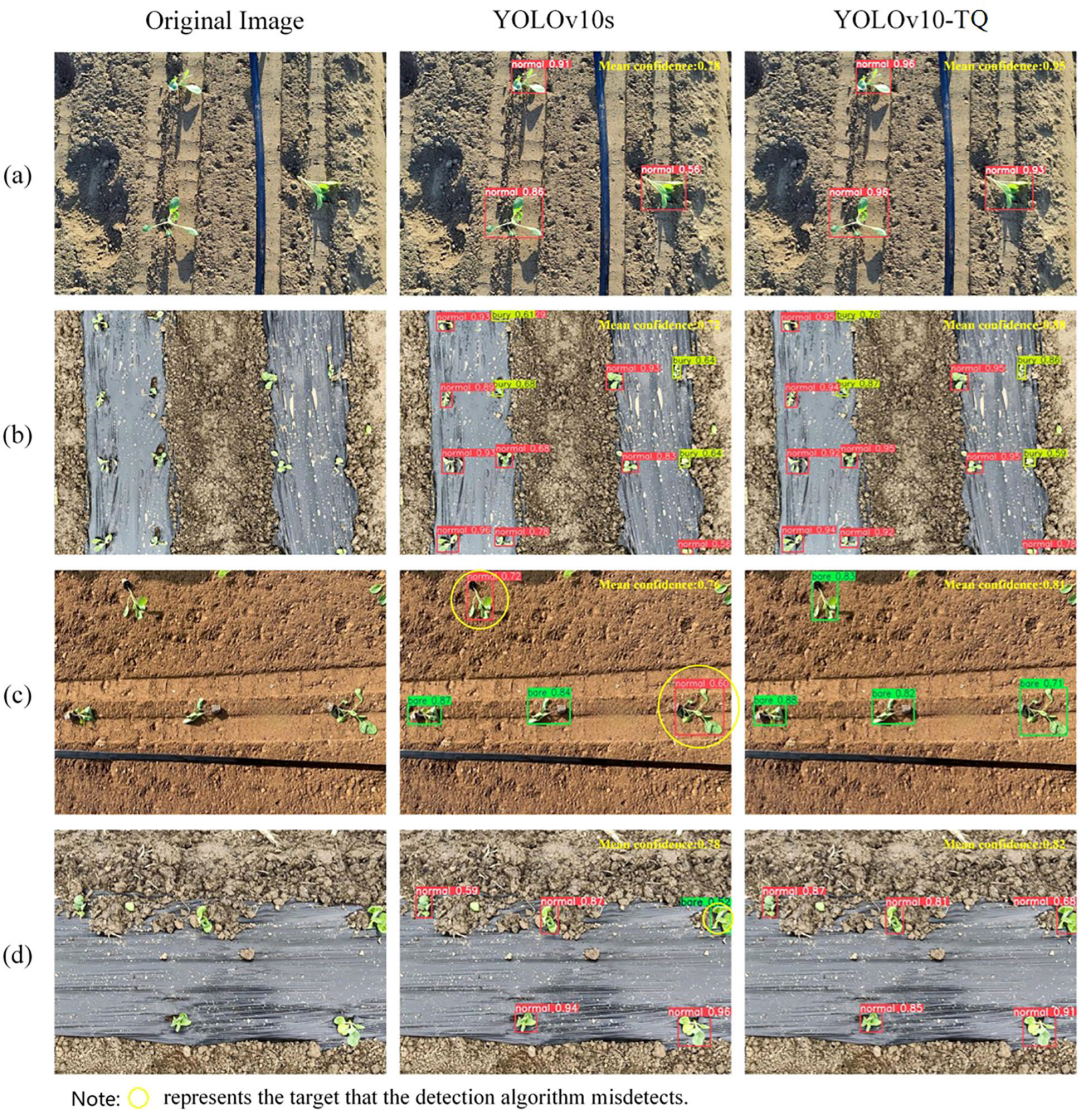
**(a)** Normal soil environment **(b)** Normal mulched soil environment **(c)** Relatively dry soil environment **(d)** Relatively dry mulched soil environment.

To validate the effectiveness of the proposed method in the actual detection of cabbage transplanting states, this study tests the YOLOv10-TQ model alongside the original YOLOv10s model on the same test set and records the average confidence score for each image. The detection results are shown in **Figure 8**. **Figures 8a, b** demonstrate that both YOLOv10s and YOLOv10-TD correctly detect transplanted states, though YOLOv10-TD exhibits superior detection accuracy. From the perspective of the average confidence score for individual images, the YOLOv10-TQ model outperforms the original model in all four detection groups, indicating that the improved model exhibits higher overall accuracy in detection tasks. In terms of class detection, **Figure 8c** shows that YOLOv10s suffers from a significant misdetection, classifying bare-root cabbage as normal state. This is because when the substrate of the cabbage seedlings is relatively dry, it closely resembles the surrounding soil environment, making detection challenging and prone to misclassification. In **Figure 8d**, the original model mistakenly classifies the normal state as bare-root. This error occurs because, under the mulching condition, the soil blocks displaced by the transplanting machine, when not influenced by the surrounding soil, are easily mistaken for the cabbage root substrate. As the substrate is a key feature of the bare-root state, this results in the misclassification of normal cabbage as bare-root. In contrast, the YOLOv10-TQ model demonstrates superior performance in these complex scenarios, successfully reducing the misclassification rate and improving detection accuracy by minimizing both false positives and false negatives.

### 3.7 DeepSORT feature extraction network retraining

**Figure 9:**
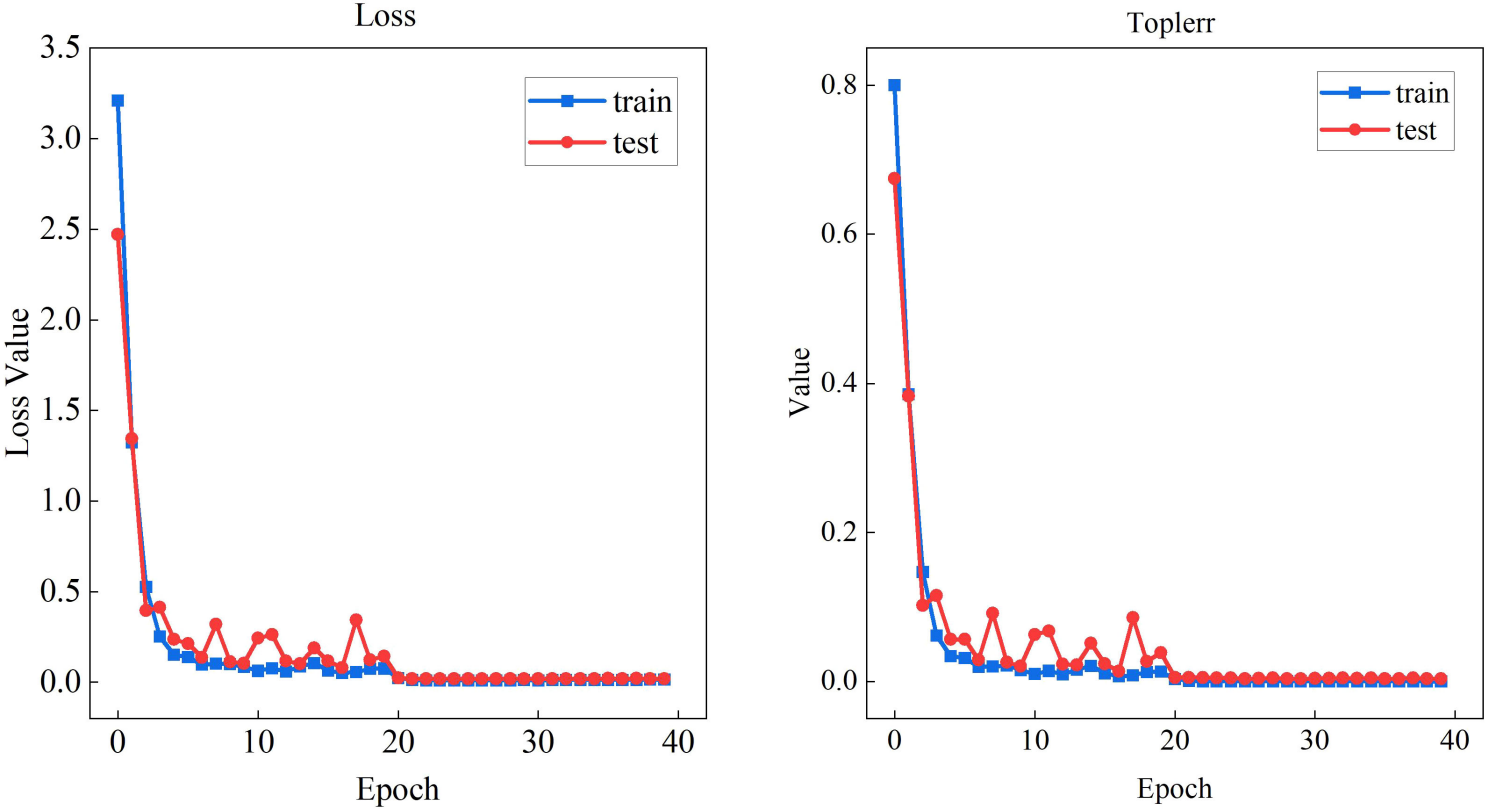
MobileViT XXS feature extraction loss and top1err convergence.

The cabbage tracking dataset was input into the improved feature extraction network for retraining. Since the tracking task is solely used for extracting feature encodings of the cabbage, the downstream classification tasks of the MobileViT XXS network are not discussed. The training loss and validation loss are recorded in **Figure 9**, where “loss” represents the total loss across all categories, and “top1err” corresponds to the highest loss for a specific category. After 20 epochs of training using the MobileViT XXS network, the model reached near convergence, with the loss function stabilizing and accuracy reaching approximately 97%. This indicates that the network has successfully learned most of the cabbage target’s appearance features.

### 3.8 Tracking ablation experiment

To evaluate the performance of the improved algorithm in cabbage transplant state counting, ablation experiments were conducted on a self-built cabbage video dataset. The experiments compared the changes in model performance with two stages of improvement. As shown in **Table 3**, the combination of the YOLOv10s-TQ detection network and the improved DeepSort tracker achieves the lowest error metrics, with MAE of 2 and RMSE of 2.2, indicating a minimal deviation between the predicted and true values, strong handling of large errors, and stable counting performance. In terms of model weight, the replacement of the original feature extraction method with the MobileViT XXS feature network resulted in the improved DeepSort tracker having a weight of only 4.17MB. This represents a reduction of approximately 90.5% compared to the original DeepSort’s weight of 43.9MB, significantly reducing model complexity and storage requirements. The precise feature capture by the YOLOv10-TQ detection network, combined with the optimized feature extraction and lower model weight of the improved DeepSort tracker, collaboratively enhances the cabbage transplant state counting performance.

**Table 3:** Comparison results of different detector-tracker combinations.

Detector	Tracker	Normal	Barried seedling	Bare root	Total count	Missed detection	MAE	RMSE	Weights (MB)
YOLOv10s	DeepSort	109	15	5	129	19	7	8.1	43.9
YOLOv10-TQ	DeepSort	114	14	9	137	11	44	4.7	43.9
YOLOv10s	Improved DeepSort	115	10	8	133	15	5.7	5.8	4.17
YOLOv10-TQ	Improved DeepSort	119	16	9	144	4	2	2.2	4.17

### 3.9 Transplant state counting accuracy comparison

**Figure 10:**
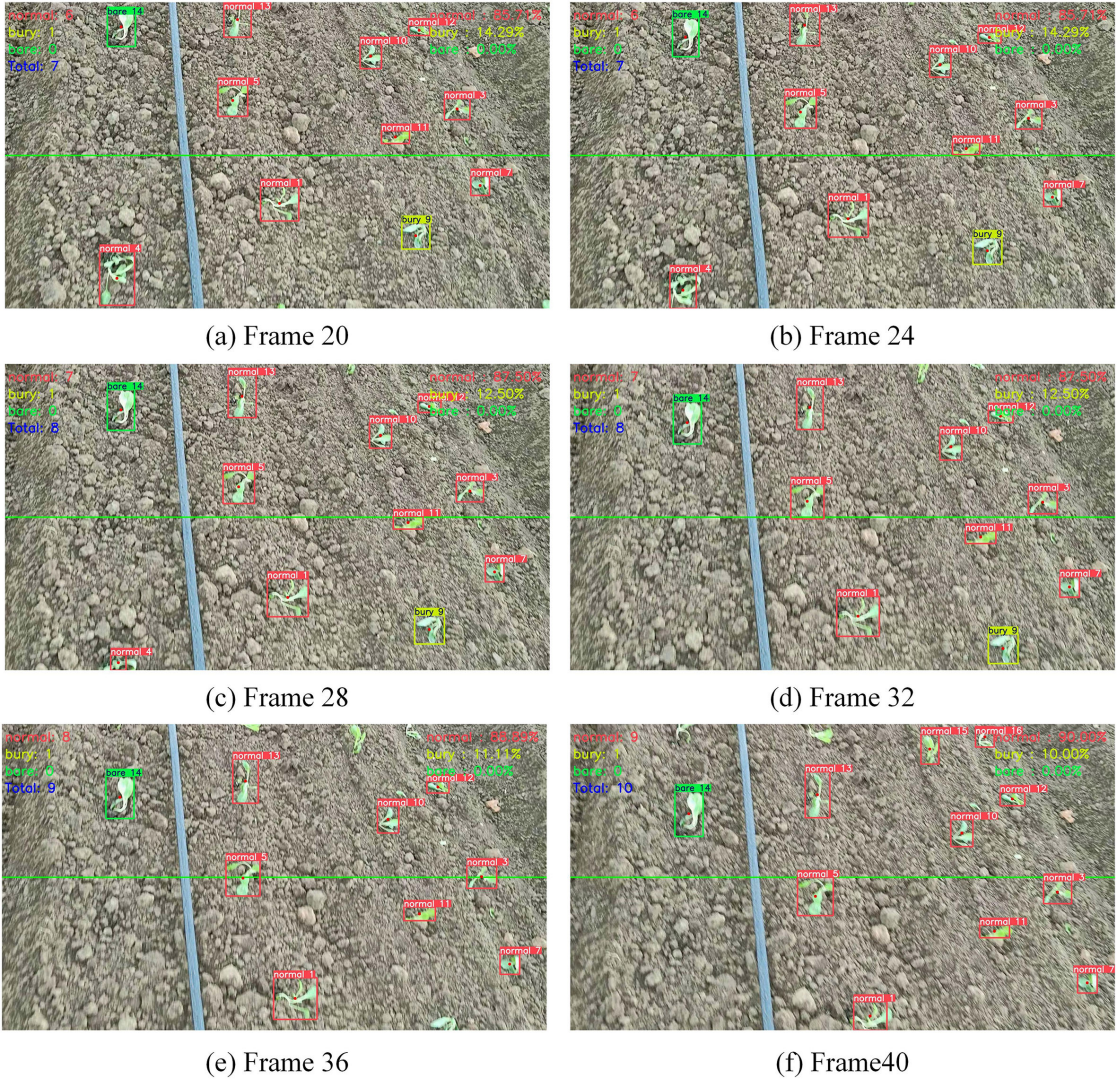
**(a–f)** Frames 20, 24, 28, 32, 36, and 40 from the same video, respectively.

To accurately estimate the number of cabbage transplant states in the video and assess the quality of transplant operations, a line-crossing counting method was employed for cabbage transplant state counting in the video. The method was tested on four manually captured cabbage videos, with a tracking example shown in **Figure 10**. When a cabbage target crosses the set line, the count increases by 1, and the counting result is displayed in the top left corner of the frame. Meanwhile, the cabbage transplant rates, including the buried seedling rate and bare root rate, are shown in the top right corner of the frame.

The number of cabbage transplant states obtained using the YOLOv10-TQ model and the improved DeepSort method was compared with the manually estimated number of cabbage transplant states, as shown in **Table 4**. The experimental results indicate that the proposed counting method yields values close to the manual estimations, with an average counting accuracy of 97.8% across the four videos. This demonstrates the applicability of the proposed counting strategy for cabbage transplant state enumeration.

**Table 4:** Testing results of different cabbage video data.

Video id	Category	Manual count (plants)	Proposed method (plants)	MCA (%)	RMSE
	Normal	169	163	96.4	
1	Buried Seedling	18	20	90	3.7
	Bare Root	9	8	88.9	
	Total	196	191	97.4	–
	Normal	32	32	100	
2	Buried Seedling	1	1	100	0.58
	Bare Root	0	0	–	
	Total	33	33	100	–
	Normal	59	59	100	
3	Buried Seedling	8	8	100	1.15
	Bare Root	17	15	88.2	
	Total	84	82	97.6	
	Normal	149	139	93.2	
4	Buried Seedling	27	31	87.1	6.24
	Bare Root	9	8	88.9	
	Total	185	178	96.2	–

**Table 5** presents the results of the single-class performance for the four videos mentioned earlier. As shown in **Table 5**, the average counting accuracy (MCA) for the normal cabbage transplant state is the highest, while the MCA for the bare root state is the lowest at 88.7%. However, the MAE and RMSE for the normal transplant state are higher compared to the buried root and bare root states, indicating a certain level of deviation between the counting results and the ground truth for the normal transplant state. In the case of normal transplant states, cabbage plants exhibit relatively consistent growth patterns, and since the number of samples for this state is the highest, the model is trained on it more frequently. In theory, this should facilitate easier detection and counting. However, in the actual data collection process, various factors such as exposure to strong sunlight prior to transplantation, causing wilting or other unfavorable conditions, affect the plants, leading to deviations during the recognition process and ultimately impacting the counting accuracy, which results in higher MAE and RMSE values.

**Table 5:** Counting accuracy of different transplant states.

Category	MCA (%)	MAE	RMSE
Normal	97.4	4	5.83
Buried Seedling	94.3	1.5	2.24
Bare Root	88.7	1	1.23

For the bare root state, although the number of samples during training is the lowest, and the model is exposed to this state less frequently, the MAE is 1 and the RMSE is 1.23, which are relatively low. The distinct features of bare root cabbage make it easier for the model to detect, thus resulting in smaller absolute and root mean square errors. However, the overall average counting accuracy (MCA) remains lower. Based on this analysis, to further improve the model’s counting accuracy for different transplant states, it is recommended to increase the number of bare root cabbage samples in future studies to enhance the model’s learning frequency for this state. During the experimental process, it was observed that the proposed method tends to overcount the buried root state compared to manual statistics. This is primarily due to leaf drop occurring during transplantation, which, from certain angles, closely resembles the buried root state, thus leading to an increased count for buried roots. Nonetheless, this discrepancy remains within an acceptable range, making the results suitable for assessing transplant quality. Overall, the proposed method demonstrates an average counting accuracy (MCA) of over 88.7% for all cabbage transplant states, 604 ensuring high precision and effective counting of various transplant states.

To enhance the statistical reliability and robustness of the model evaluation, we have conducted 5-fold cross-validation on the full dataset. In this setting, each fold served once as the validation set while the remaining four were used for training, ensuring comprehensive coverage of all samples.

The results of the cross-validation were acquired, where the mean and standard deviation (mean ± SD) are provided for key metrics. The average mAP@0.5 achieved 86.1 ± 0.4%, and MCA reached 97.5 ± 0.3%, indicating that the model maintains stable performance across all folds with minimal variance. It confirmed that the proposed model generalizes well to unseen field scenarios despite the moderate dataset size. And the results could strengthen the experimental validation and support the claims regarding the system’s robustness and consistency.

**Figure 11:**
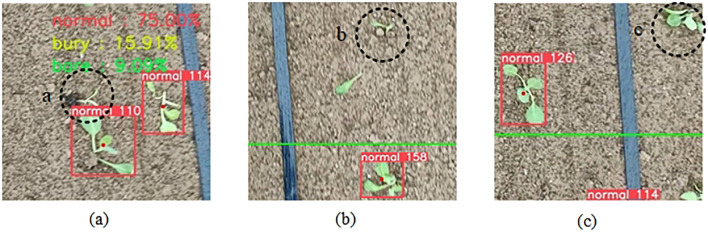
The representative failure cases highlighted by black dashed circles. **(a)** Counting errors due to overlapping seedlings. **(b)** Seedlings undetected when occluded by soil clods. **(c)** Edge blurring at image boundaries.

Several representative failure examples with black dashed circles are shown in **Figure 11**. Three major failure modes including counting errors due to overlapping seedlings, seedlings undetected when occluded by soil clods and edge blurring at image boundaries. The occurrence frequency of these failure cases is primarily influenced by environmental illumination, soil surface roughness, and camera installation angle, which jointly affect image contrast and feature separability. To mitigate these three types of failure cases, several strategies can be adopted. Introducing multi-angle or stereo imaging can effectively reduce counting errors caused by overlapping seedlings by providing additional depth cues. Employing adaptive illumination correction and texture enhancement can improve the visibility of seedlings partially occluded by soil clods. And integrating a boundary-aware refinement module with lightweight deblurring algorithms at the image periphery can restore edge sharpness and ensure more accurate detection near image boundaries. These improvements together would enhance model robustness and reliability under complex field conditions.

## 4 Design of the cabbage transplanting status visualization system

Building on the aforementioned research, a cabbage transplanting status visualization system was developed. The system consists of two main modules: the cabbage transplanting status detection module and the cabbage transplanting status counting and evaluation module, aimed at improving precision in agricultural monitoring and decision-making.

**Figure 12:**
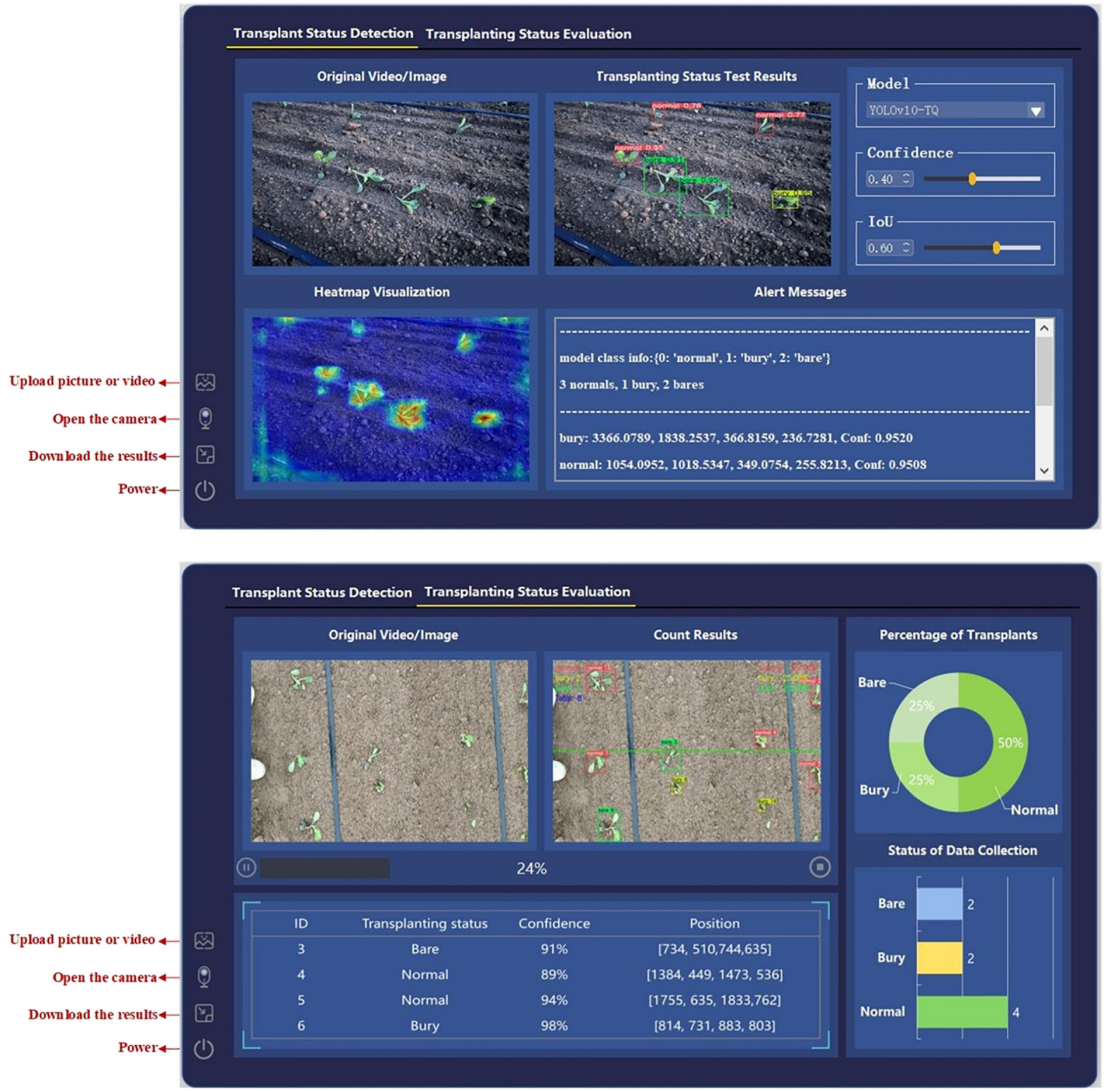
Cabbage transplanting detection and evaluation interfaces. **(a)** Transplanting status detection interface. **(b)** Counting and evaluation interface.

For the system, the data input mechanism supports two operational modes: one is real-time detection via direct camera activation, which is tailored for real-world deployment on transplanters; the other is offline import of images or videos, which is suited for *post-hoc* assessment of transplanting quality. The performance evaluation result export module is capable of generating key metrics, including RMSE, MAE, and MC for multiple models, facilitating comprehensive comparative analysis. As shown in **Figures 12a, b**, both data input and result export operations can be executed directly through intuitive interactions within the “Transplant Status Detection” and “Transplanting Status Evaluation” interfaces, ensuring operational efficiency and accessibility.

### 4.1 Cabbage transplanting status detection module

The detection module interface is shown in **Figure 12a**. The interface design consists of several sections, including the model configuration area, data loading area, detection result display area, heatmap visualization area, and detection result information prompt area. Within this module, users can not only select the model but also adjust its parameters. The detection result information prompt area provides detailed information about the transplanting status detected in the image, such as the count of each category in a single frame/image, the coordinates (xywh) of each target, and the associated confidence scores. The heatmap visualization area enables class activation map analysis of the detected image, generating high-resolution heatmaps using the Grad-CAM++ algorithm. This allows for a clear representation of the neural network’s focus on key features of the cabbage seedlings. This feature is particularly useful for users to validate the model’s decision-making, especially in complex scenarios with abnormal transplanting statuses, where the spatial correlation between the heatmap distribution and detection boxes aids in verifying the algorithm’s credibility.

### 4.2 Cabbage transplanting status quality evaluation module

The interface of the cabbage transplanting status quality evaluation module is shown in **Figure 12b**. The system is structured into three main components: a data loading section, a counting result display area, and a quantitative visualization panel. A dual-view layout is adopted to simultaneously present the original images or video streams alongside their corresponding detection and counting results, enabling users to intuitively compare raw and processed data for real-time monitoring and accuracy verification.

In the top-left corner of the detection results, the system clearly marks the three transplanting status categories used for line-crossing counting, allowing users to track the count for each category in real time. A progress bar displays the current processing status, facilitating user awareness of ongoing analysis. Additionally, a detailed record panel lists each counted target’s ID, transplanting status, confidence score, and detection coordinates, providing comprehensive real-time information.

The quantitative visualization area illustrates the proportion of each transplanting status, including the bare-root rate, buried seedling rate, and normal rate, using a pie chart to represent the overall distribution of transplanting quality. In addition, a horizontal bar chart displays the absolute count of each category, providing a clear and data-driven foundation for evaluating transplanting performance and supporting informed decision-making in precision agriculture.

Under the environment of RTX 3090 GPU (24 GB memory) with Python 3.8.0 and PyTorch 1.9.0, the system achieves an average frame rate of 43.5 ± 2.1 FPS for the end-to-end full process (including image preprocessing, YOLOv10-TQ detection, improved DeepSort tracking, and result visualization), with a stable single-frame processing latency of 23.0 ± 1.0 ms. Specifically, the inference time of the detection and tracking module is 17.7 ± 0.7 ms, which is 20.6% faster than the baseline model without losing accuracy (mAP remains 86.3%).

## 5 Conclusions

To address the challenges of state recognition difficulties and large counting errors in cabbage transplant operations, an intelligent detection and transplant quality evaluation method is proposed and a cabbage transplanting status visualization system is designed, which integrates an improved YOLOv10 detection network with a lightweight DeepSort tracking algorithm. The method is described as follows:

1. To address the inefficiencies of traditional manual evaluation of vegetable transplanting and the insufficient accuracy of mechanized transplant quality detection, a cabbage transplant state detection method based on the improved YOLOv10 (YOLOv10-TQ) is proposed. The method introduces a Triplet Attention mechanism to strengthen multi-dimensional feature interaction capabilities, combined with a QFocalLoss and cross-entropy joint loss function to optimize the issue of class imbalance. Experimental results show that the proposed YOLOv10-TQ algorithm achieves a mean Average Precision (mAP) of 86.3% for detecting normal, buried, and bare-root states, representing a 1.8% improvement over the baseline model. With only 8.1M parameters, YOLOv10-TQ achieves a significant balance between accuracy and efficiency compared to mainstream algorithms such as YOLOv11 and RT-DETR.
2. To address the challenge of precise counting of crop transplant states in complex field environments, a lightweight DeepSort tracking structure is designed. First, the differences in feature extraction ratios between pedestrians and cabbage are analyzed, and a tailored feature extraction ratio for cabbage is developed. Next, the MobileViT XXS is used to replace the original feature extraction network, reducing the number of parameters by 90.5%. Finally, a line-crossing counting mechanism is introduced to accurately associate cabbage targets across frames.
3. After integrating the detection and tracking modules, a complete transplant state recognition and counting system is constructed. On the self-built dataset, the system achieves an average counting accuracy of 97.8%, with MAE and RMSE reduced to 2 and 2.2, respectively, providing quantitative support for the intelligent quality assessment of transplant operations in agricultural production.

## 6 Future works

While the proposed method achieves efficient and accurate detection and evaluation of cabbage transplanting status, it still has room for improvement in adapting to more complex agricultural scenarios and practical application requirements. Future work will focus on the following aspects to further enhance the method’s robustness and practicality:

(1) Expanding the dataset

Due to advancements in transplanting technology, samples of abnormal statuses (buried seedlings, bare-root seedlings) are far fewer than normal ones, leading to significant class imbalance. Though data augmentation and loss function optimization have mitigated this issue, the model’s accuracy and generalization for abnormal statuses remain limited. Future work will focus on expanding the dataset with samples from diverse regions, cabbage varieties, and extreme scenarios (e.g., severe soil compaction, heavy rainfall) to enhance feature learning and adaptability.

(2) Real-time monitoring deployment and *post-hoc* Evaluation Integration

The system proposed in this work performs *post-hoc* quality assessment based on images and videos captured by external devices. It should be mentioned that the whole system can be designed primarily for real-time, in-field monitoring and secondarily for *post-hoc* evaluation. On machine, the pipeline is: detection → tracking → over-line counting → metric display. The interface reports bare-root, buried, and normal percentages in real time to support same-shift correction. *Post-hoc* analysis batch-processes recorded videos or images for audit and traceability. This choice is supported by: (i) an architecture with camera streaming, edge inference, and immediate UI display; (ii) a GUI that accepts both live camera feeds and offline files; and (iii) lightweight models suitable for edge devices. The detection and counting modules can be deployed on an edge-computing unit mounted on the Kubota 2ZSY-4 vegetable transplanter.

When the proposed models are deployed on the transplanter, vehicle jolting and mechanical vibration during operation may cause relative displacement between the camera and the cabbage targets during the camera’s exposure period, resulting in motion blur, which manifested as diffused target edges and reduced detail contrast. This phenomenon interferes with the model’s feature extraction process, leading to a decrease in localization confidence and recall rate for transplanting-state detection, ultimately reducing the accuracy of operational quality assessment.

This issue can be addressed through a “hardware compensation–software optimization” collaborative strategy. On the hardware side, a lightweight three-axis electronic gimbal is integrated at the camera deployment end of the transplanter. By leveraging real-time attitude sensing and dynamic compensation, the system suppresses relative displacement between the camera and target, thereby eliminating the root cause of motion blur at the hardware level. On the software side, an adaptive motion-blur kernel estimation module and a lightweight deblurring submodule are embedded at the front end of the detection–counting model to restore target edge sharpness and detail contrast. Together, these measures ensure high localization confidence and recall rates for transplanting-state detection under motion-blur conditions, supporting accurate evaluation of operational quality.

(3) Optimizing the user interface

The current system provides intuitive functions (real-time camera detection, offline image/video analysis) but can be further improved. Future efforts will refine interactive design for simpler operation, enrich data visualization (e.g., dynamic quality trends, abnormal status heatmaps) for clearer insights, and introduce intelligent recommendation modules to adjust monitoring strategies based on crop traits and environmental conditions, boosting practicality.

## Data Availability

The raw data supporting the conclusions of this article will be made available by the authors, without undue reservation.
